# Maintenance of Fish Health in Aquaculture: Review of Epidemiological Approaches for Prevention and Control of Infectious Disease of Fish

**DOI:** 10.1155/2018/5432497

**Published:** 2018-02-26

**Authors:** Ayalew Assefa, Fufa Abunna

**Affiliations:** ^1^Sekota Dryland Agricultural Research Center, P.O. Box 62, Sekota, Ethiopia; ^2^College of Veterinary Medicine and Agriculture, Addis Ababa University, P.O. Box 34, Bishoftu, Oromia, Ethiopia

## Abstract

Aquaculture is rapidly growing part of agriculture worldwide. It makes up around 44 percent of total fish production globally. This increased growth of production is achieved despite facing many challenges in the aquaculture environment. Among production limiting challenges, the infectious disease takes the lion share by causing multibillion-dollar loss annually. To reduce the impact of the fish disease, it is necessary to address health constraints based on scientifically proven and recommended ways. This review aims at pointing out some of the best approaches to prevention and control of infectious disease in aquaculture. Among the effective prevention and control strategies, vaccination is one of the key practices. Types of vaccines for use in fish include killed vaccines, attenuated vaccines, DNA vaccines, recombinant technology vaccines, and synthetic peptide vaccines. Administration techniques of vaccines in fish include oral, injection, or immersion methods. Antibiotics are also in use in aquaculture despite their side effects in the development of drug resistance by microorganisms. Biological and chemical disease control strategies such as using probiotics, prebiotics, and medicinal plants are widely in use. Biosecurity measures in aquaculture can keep the safety of a facility from certain disease-causing agents that are absent in particular system. Farm-level biosecurity measures include strict quarantine measures, egg disinfection, traffic control, water treatments, clean feed, and disposal of mortalities. In conclusion, rather than trying to treat every disease case, it advisable to follow a preventive approach before the event of any disease outbreaks.

## 1. Introduction

Fisheries play a great role in food security and livelihood and are a source of income and social development in developing countries [1]. Recently the sector attracted great attention and it is growing rapidly through the development of aquaculture [2]. New technological advances and increased demands for fish as a source of animal protein are the main reasons for the industry's growth. Because of expansion of the industry, the culture methods have become more intensive for producing higher yields [3]. Aquaculture production of fish makes up forty-four percent of total fish production in 2014 which is 74 million tons of fish worth of 160 billion dollars. Almost all fish produced from aquaculture is for human consumption [1].

Huge loss of production in aquaculture is occurring because of many reasons. Among these causes, a disease is the most serious constraint that causes damage to the livelihood of farmers, loss of job, reduced incomes, and food insecurity. Studies showed that almost fifty percent of production loss is because of diseases which are more severe in developing countries. This is because ninety percent of the aquaculture firm is in the developing world. The annual loss of revenues because of disease reaches up to 6 billion dollars. For instance, in Chile, infectious salmon anemia alone costs 2 billion dollars and caused 20000 workers to lose their jobs. In China, one of the leading countries in aquaculture production has a loss of 15 percent of the total fish production to diseases [4].

To overcome losses because of infectious diseases in aquaculture, it is necessary to act upon every health constraint based on scientifically proven and recommended as well as locally applicable ways. Challenges in aquaculture because of climate change, limited water sources, and the growth drive the need for epidemiological approaches in keeping aquatic animal health safe [5]. As “prevention is better than treatment,” it is advisable to focus on preventing the occurrence of disease rather than treating it [6]. The uses of improved husbandry/management practices, movement restrictions, genetically resistant stock, dietary supplements, nonspecific immunostimulants, vaccine, probiotics prebiotics, medicinal plant products, water disinfection biological control, antimicrobial compounds, water disinfection, and control of movement are the best approaches in control of infectious diseases of fish [7].

The use of antibiotics is under strict control and regulatory measures because of drug resistance and residue related issues [8]. In response to reduced antibiotic use in fish, production vaccines have been playing a key role in infectious disease control in aquaculture for decades. Vaccines get wide acceptance for the fact there is no risk of drug resistance development in vaccinated animals and protection of minority unvaccinated animals because of herd immunity [9].

A single approach to prevention and control of aquaculture health is not successful alone. Rather a combination of different strategies is effective. Setting up a national or regional information exchange between farmers and responsible parties is compulsory. Besides applying all these strategies, surveillance for diseases and having sensitive and specific diagnostic tests are invaluable to assure healthy fish. This review has the aim of summarizing some of the best approaches to prevention and control of infectious disease of fish in an aquaculture environment.

## 2. The Role of Vaccines in Control and Prevention of Infectious Disease in Aquaculture

Advancing vaccination is one of the most important, and probably the priority, approaches to prevention and control of infectious disease of fish. Treating many of the bacterial infections in aquatic animals using antimicrobials only is impossible [10]. There are improvements in fish vaccination recently. Some of the improvements include immunization of large stock at a time and the development of multivalent vaccines [11]. Vaccination is widely in use in almost all food-producing animals. In aquaculture, it reduces the use of antibiotics and protects fish from infectious diseases. It also avoids the risk of drug resistance. Protection at stock level because of herd immunity can be achieved and the need for licensing and registration of new vaccine is much easier than antibiotics [11].

There are few important considerations that should be taken into account before application of vaccination in fish. These considerations include the following points: fish species to be vaccinated, status of the immune system of the fish, production cycle, and life history of the aquaculture system, which diseases need to control in aquaculture, when do these diseases occur (seasonal distribution of diseases in the aquarium), farming technology (handling and mechanization), environment (temperature and salinity), stress factors, nutrition, and cost benefit [12].

### 2.1. Historical Overview of Fish Vaccination

Fish vaccination was started by vaccinating against* Aeromonas salmonicida* infection in Cutthroat in 1942 [13]. Currently, vaccines in use are the conventional type of vaccines because of lack of advanced information on the immunology of fish. Vaccines available are oil adjuvant, injectable vaccines [13]. The salmon genome is now fully sequenced [14], the genome and several other fish species as well. These findings can lead to novel vaccine development strategies in near future [15]. Vaccines against intracellular bacterial and viral pathogens will be one of the big challenges for the coming years. DNA vaccine will play a role in such cases [13].

### 2.2. Types of Fish Vaccines

Modern vaccines can be classified as killed, attenuated, DNA, synthetic peptide, recombinant vector, genetically modified, and subunit vaccines. Whole organism vaccines showed a better advantage than other types of vaccines. However, most of the vaccines do not completely prevent disease [10].

The antigens are weak in most conventional vaccines that they cannot induce immunity in the recipient. In addition, they may not be easy for development to prevent emerging pathogens, the presence of antigenic shift and antigenic drift, during immune evasion of the host by pathogenic organisms, and microbes which cannot be grown by in vitro propagation, and development of these vaccines is a slow and time-consuming process, which sometimes poses difficulty in timely countering of emerging and reemerging pathogens. That is why advanced technologies of vaccine designing strategies are developed for the discovery of newer types of effective vaccines [16].

#### 2.2.1. Killed Vaccines

Killed vaccines are conventional types of vaccines prepared by killing the infectious agent and using it as an antigen to induce an immune response. Most of the commercial vaccines currently used in aquaculture are killed vaccine types. The advantages of these vaccines are as follows: they are easy to design, are stable in storage, and are less expensive and there are no virulence issues [17]. Preparation strategy of these vaccines is that they most of the time target the outer surface of microorganisms or inner parts without avoiding the ability to replicate when administered to the host [12]. Infectious hematopoietic necrosis virus,* A. salmonicida*, and* V. salmonicida* are some diseases that can be prevented by killed vaccines.

#### 2.2.2. Attenuated Vaccines

These are also conventional vaccines which are used in food-producing animals and humans to prevent disease [18]. They are prepared by repeated laboratory passage, physical and chemical attenuation of the organisms to lose their virulence without killing them. Laboratory studies have shown the effectiveness of live vaccines in fish. They induce mucosal, cellular, and humoral immunity [19]. Attenuated organism replicates in the target host without any clinical signs [20].

#### 2.2.3. Deoxyribonucleic Acid (DNA) Vaccines

They are a recent type of vaccines which are the result of the advancement of molecular biology. To develop a DNA vaccine, there is no need to use the antigen as a vaccine, rather the gene that code for the antigen is taken by molecular techniques and can be given as a vaccine [21]. DNA vaccines contain one or more genes of a pathogen. Intramuscular injection of these vaccines confers immediate and a durable protection from diseases in farmed salmonids against economically important diseases such as infectious hematopoietic necrosis virus [22] and viral hemorrhagic septicemia virus [23] which were controlled by DNA vaccines.

DNA vaccine is designed first by identifying and cloning a protective antigen from the pathogen. For example, for some pathogenic viruses of fish like VHSV and IHNV, protective antibodies are known to be against surface glycoprotein of the viruses. Therefore, the glycoprotein gene and the regulatory sequences that allow expression in eukaryotic cells was an option for the development of DNA vaccines. To administer as a vaccine the plasmid is produced in bacterial culture, purified, and quality-assured. After this process, a DNA vaccine will be administered and taken by cells of the host to produce the glycoprotein. This situation leads to detection of the antigen by the immune system of the fish [24].

DNA vaccines in fish have been well studied for salmonid rhabdoviruses IHNV and VHSV. These vaccines have been found to be effective in salmon aquaculture industry to reduce the impact of these viruses. In addition, the two virus DNA vaccines have been also tried for other viral diseases of fish like spring viraemia of carp virus and hirame rhabdovirus. Even though most of DNA vaccines have been developed for viral diseases of fish, DNA vaccine was tried to prevent bacterial kidney diseases of salmon caused by* Renibacterium salmoninarum*. However, this vaccine was not found to be effective [24].

The advantage of a DNA vaccine is that it is based on purified plasmid DNA carrying only a single gene from the pathogen, which makes it noninfectious and is unable to replicate within the host, there is no risk of transferring the actual disease with the vaccine. That is why DNA vaccines are considered safer than conventional vaccines, that is, inactivating the whole virus, with or without oil adjuvant, or attenuated live virus. These vaccines do not use adjuvants to administer like that of conventional vaccines which make them free of postvaccination side effects. In addition, all these DNA vaccines do not contain unknown impurities commonly found in whole organism types of vaccines [24].

#### 2.2.4. Recombinant Vector Vaccines

Recombinant vector vaccines are the result of biotechnological advancement prepared by taking only the immunogenic regions of a pathogen and expressing it in a heterologous host. The immunogenic part of the organism taken and expressed to carriers. The proteins are then produced in large quantities in vitro and then purified for use as a vaccine. Being easy to produce in large quantities of proteins and effectively expressing the antigenic protein are the main factors considered during vector selection. Infectious salmon anemia and infectious heamatopoetic necrosis disease viruses have been expressed in vectors as a vaccine to protect salmon [10].

#### 2.2.5. Subunit Vaccines

When culturing of the organism is difficult, these vaccines become useful by taking immunogenic part and using it as a vaccine. Subunit vaccines are safe for use but their immunogenic nature is very poor compared to inactivated, whole organism vaccines. Adjuvants are therefore needed to improve immunogenicity [10].

#### 2.2.6. Genetically Modified Vaccines

In vitro passaging of organisms results in a build-up of genome mutations that make the organism weaken. Genetically, microorganisms may be attenuated by molecular approaches that involve removal of genes responsible for its pathogenicity. Live attenuated vaccine will replicate to a lower titer and can stimulate humoral and cellular immunity.* Aeromonas salmonicida* in salmon can be prevented by these preparations [10].

#### 2.2.7. Synthetic Peptide Vaccines

These types of vaccines are produced from short sequences of amino acids prepared synthetically to act as antigens [17]. These can be used as a suitable antigenic site. Studies showed that vaccinating fish with peptides is less practicable because of lack of understanding the fish immune response to different antigens and being not potent enough and thus requires a carrier molecule [25]. These vaccines have been in use as prevention of infectious disease like nodavirus, viral hemorrhagic septicaemia, rhabdovirus, and birnavirus [10].

### 2.3. Methods of Administering Vaccines to Fish

Successful vaccination depends upon both the development of protective vaccines and their correct use [20]. Besides deciding which diseases to vaccinate is necessary to clearly understand how to administer vaccines and when to administer a booster dose (booster dose) must be considered. For best protection, vaccination should be carried out sometime before exposure to the pathogen, to give immunity plenty of time to develop. Water temperature may be an important factor when deciding when to vaccinate, as well as the size of fish, being the major feature regulating the development of immune competence [26].

#### 2.3.1. Oral Vaccination

Oral vaccination is easy to apply and avoids stress in fish. The vaccine is incorporated to the feed during production, or it may be coated with pellets or encapsulated [20]. Oral vaccination is recommended for secondary or booster vaccinations [10]. Disadvantages of oral vaccination include that it may not give a uniform protection and requires a large dose, and it may have additional cost of encapsulation [26].

#### 2.3.2. Immersion Vaccination

When applying immersion, vaccines are applied to the surfaces of the fish. The antigen uptake is via the gills, the skin, and the lateral line. The fish may be dipped for a short period of time in concentrated vaccine solution. The vaccine solution may also be sprayed onto the fish. Immersion vaccination is particularly convenient for small fish and fingerlings, which are impractical to handle for injection; the other advantage is that it causes minimal stress in fish; a solution of vaccine can be reused. Its disadvantages are it is labor-intensive and it is expensive to buy tanks and specialized equipment is required [10].

#### 2.3.3. Injection Vaccination

Vaccination by injection is the delivery method generally resulting in best protection and is the only choice for adjuvant vaccines [8]. The advantages of injecting a vaccine are attaining high protection and needing relatively minimal dose because correct dosage calculation is easy and economical for larger fish, and a multivalent vaccine can be administered. The disadvantages of this administration are as follows: not suitable for small fishes, adhesion formation, stress in fish and feed intake reduction, damage during injection which may cause multiple deaths in fish, and not being administered in very young stock due to immunity development may not sufficient [20].

#### 2.3.4. Commercially Available Vaccines Currently

A number of vaccines have been approved for use in aquaculture currently worldwide (Table 1).

## 3. The Use of Antibiotics in Aquaculture

Currently, there is a common understanding that antibiotic for protecting fish from disease should remain low [27]. Extensive use of antibiotics may result in resistance development. The use of antibiotics in aquaculture is no more primary treatment option. Even though using antibacterial agents in food animal species, including fish, is controlled by regulations, particularly in Europe and the USA, a wide range of medically and veterinary inhibitory compounds are in use in aquaculture [28]. Administering antimicrobials in aquaculture is different from administering in terrestrial animals. This difference particularly adding drugs to the water with or without feeds results in environmental disturbance of the microbiota [29]. Using antibiotics in fisheries routinely in aquaculture that are in use for human medication is a risky act. Aquaculturists are advised to use other prevention approaches rather than antibiotics administering but if the use antibiotics is a must in some circumstances, they have to administer only approved drugs for fish [30].

The American FDA developed a list of chemicals and antibiotics list for use in aquaculture which has undergone review and is classed as new animal drugs of low regulatory priority. These include compounds such as acetic acid, carbon dioxide gas, hydrogen, sodium chloride, or even garlic (used for control of helminth and sea lice infestations of marine salmonids), onion (used to treat external crustacean parasites), and the ice used to reduce the metabolic rate of fish during transport [31].

### 3.1. Administration Strategies of Antibiotics in Aquaculture

#### 3.1.1. Medicated Feed (Oral Administration)

Medicated feed is one of the successful methods of administering drugs in aquaculture. The careful administration of drugs is necessary because some of the causes of disease like stress lead to treatment failures. Fishes can be stressed in cases of increased fish density, poor or inadequate nutrition, poor water quality, parasite infestation, and handling [30].

#### 3.1.2. Injection

In case of severe infections, it is advisable to use injection for effective action than medicated feed. It is advisable to use this administration technique for a valuable individual such as ornamental fish because it is labor-intensive and time-consuming. Injection ensures immediate effect by reaching blood quickly. Injection sites include the intraperitoneal cavity and the intramuscular sites [32].

#### 3.1.3. Immersion

Rather than systemic infections, this type of administration strategy is recommended for external infection treatment. It has many disadvantages as follows: it requires a separate tank, it needs more antibiotics than oral administration, and strict water to drug volume ratio adjustment is required [32].

## 4. The Use Probiotics in Aquaculture

Boosting the natural defense of a fish is one of the researchable areas with many beneficial advantages [56]. The main search has been for substances which can be incorporated in feed and delivered orally to fish but others may be injected along with vaccines. Many of the early reports of commercial benefits were not supported by investigations of the mechanism of action and evidence for the involvement of the immune system could not be confirmed. More recent studies are now accompanied by data on the effects of treatment on a number of immune bioassays and, though the mode of action is unknown, there appears to be some form of immunomodulation. Whatever their action, immunostimulants directly or indirectly enhance the specific or nonspecific defense mechanisms, or both [57].

Biological disease control in aquaculture is among the best approaches in infectious disease control [57]. Probiotics are a bacterial culture of bacterial strains which are nonpathogenic to fish [58]. The other definition of probiotics is that live microorganism, administered to hosts to develop a protective immune status. After being administered to fish they multiply themselves to occupy the gut of the fish, they help normal microflora, and they maintain microbial balance in the hosts [59].

There are several criteria to be considered when choosing the suitable probiotics strain. The characteristics to consider include the following: host origin, the safety of the strain, production of antimicrobial substances, ability to stimulate host immune response, or efficient competition with pathogens for intestinal mucosa adhesion sites. One of the most common ways to get a source of these bacteria is to perform in vitro antagonism tests, in which pathogens are exposed to the candidate probiotics or their extracellular products in liquid and/or solid medium [7].

Many microorganisms have been evaluated as probiotics in aquaculture.* Bacillus subtilis*,* Lactobacillus acidophilus*,* Lactobacillus sakei*, and* Shewanella putrefaciens* are few of them. These can be used in fish and other cultured animals to prevent disease and promote weight gain. Probiotics can be applied to the feed, or they can be added to the water directly. The other administration strategy is encapsulation. Encapsulation helps by improving nutritional values and proper delivery of the microbe to the host without waste of live organisms [60].

### 4.1. Administration Strategies of Probiotics

Probiotics can be added to the feed in the water tank. Many studies have been conducted to recommend the best way of administering, optimal dose, and the technical solutions required, especially to keep the probiotics alive in dry pellets [60].

#### 4.1.1. Dietary Administration

One of the most important and probably widely applicable administration of probiotics is incorporating directly to feed pellets. Probiotics used for dietary incorporation are mainly in the form of spores. During the addition of probiotics, the viability should be checked continuously in order to confirm protective enhanced immunity in the fish. They can be added as freeze-dried cultures that can be mixed with lipids as top dressings in the feed [60].

#### 4.1.2. Microencapsulation

The other widely used probiotics administration strategy is that the application of a process called encapsulation. In this process, the cells of the organism at high density are encapsulated in a colloidal matrix using alginate, chitosan, carboxymethylcellulose, or pectin to physically and chemically protect the microorganisms [61]. Focusing on the application to aquaculture has effectively encapsulated cells of* Shewanella putrefaciens* in calcium alginate, demonstrating the survival of encapsulated probiotic cells through the gastrointestinal tract of sole* (Solea senegalensis)*. Encapsulation in alginate matrices protects bacteria from low pH and digestive enzymes [7].

#### 4.1.3. Immobilized Probiotics

Entrapment of cells released in a gel matrix of alginates around the core substance is known as the wall of immobilization. Probiotic immobilization is a new technology used extensively in the dairy and pharmaceutical industries, applied to a LAB. In particular, cell immobilization has been reported to offer many advantages for biomass and metabolite production compared with free cell systems, such as high cell [60].

## 5. The Use Prebiotics and Medicinal Plants in Aquaculture

The other important immune boosters in aquaculture are prebiotics. They are referred to as food for probiotics. They are resistant to attack by endogenous enzymes and hence can reach the site of action to promote the proliferation of gut microflora. Some of the prebiotics, that are currently used in animal feed, are mannan-oligosaccharides (MOS), fructooligosaccharide (FOS), and mixed oligo-dextran [62].

Plant product application in aquaculture for disease control is one of promising alternatives to antibiotics. They stimulate the immune system of fish, avoid stress, and act as antibacterial and antiparasitic agents due to their active chemical ingredients [63]. They can be administered by extracting their active component or the whole plant material can be added to the aquarium directly. Depending on the type of plant part used and the season of harvest of the plant material, their active ingredient may be varied so knowledge of the plant and season of the collection is necessary. Medicinal plants can be administered to fish by injection oral administration and through immersion or baths [64]. Injecting the extracted material is an effective method for large fish [65].

## 6. Biosecurity Measures in Aquaculture

Biosecurity is any management action to prevent the introduction of disease-causing agents to aquaculture facility [66]. Farm-level biosecurity measures involve the application of a combination of activities more or less which includes strict quarantine measures, sanitation of equipment, disinfection of egg, traffic control, water treatments, use clean feed, disposal of dead appropriately. These protocols should be implemented during the introduction of new stock as well as implementing them for reducing pathogens and to avoid transferring pathogens from one stock to another. Most diseases of aquaculture can be overcome by meticulous application of biosecurity measures. Stocking density reduction is one of the most important approaches to control diseases of fish in aquaculture. Low stocking densities are a very useful first step measure when ectoparasite infections break out, along with increasing water flow, to achieve a greater flushing effect on the parasites. For an interesting account of stocking density and fish welfare, see [67].

### 6.1. Quarantine and Restriction of Movement in Aquaculture

Quarantine is confining aquatic animals that are introduced from outside and they are with unknown health status before introducing to the stock. During this time strict observation of animals and using appropriate diagnostic test is required [68]. The duration of quarantine may range from fifteen days to 3 months [69]. After a correct diagnosis of a disease in question, treatment should be given with efficacious agents for the appropriate period of time. Prophylactic treatments can inhibit developing clinical signs and inappropriate use of antibiotics will lead to developing antibacterial resistance [70].

### 6.2. Disinfectants and Pesticides in Aquaculture

Disinfection involves the use of physical or chemical agents to remove microorganisms usually on inanimate objects. In aquaculture, disinfectants can also include compounds used to destroy microorganisms living on the surface of fish eggs. These agents are used in aquatic animal rearing facilities as part of biosecurity protocols to control the spread of aquatic animal pathogens [71]. The cleaning and drying of ponds properly can be phenomenal in controlling of many diseases of fish in aquaculture. A pond that has high quality clean and water well- aerated water is important in producing healthy fish and critical to those species native to oligotrophic waters such as the salmonids [72].

Quaternary ammonium compounds, formaldehyde, hydrogen peroxide, isopropyl alcohol, glucoprotamine, chlorine iodine, and iodophors, are mostly used as a disinfectant in aquaculture. Apart from being toxic to fish quaternary ammonium compounds are effective in killing organisms in inanimate objects. Chlorine can also be used but it must be neutralized adequately to avoid killing of fish. Equipment disinfected with iodine-containing compounds must also be rinsed off prior to use because they can be toxic [73].

### 6.3. Surveillance for Diseases of Fish in Aquaculture

Any aquatic health plan or any policy development for aquatic animal health is not possible without quality health data. This data can be used for disease control, quarantine, and health certification which can be achieved by conducting aquatic animal surveillance [73]. Surveillance to avoid introduction of disease is an important element of any biosecurity strategy to identify the possible route of disease introduction to aquatic firm and to detect the emergence of a new disease which will ensure that control strategies can be implemented before the pathogen becomes widespread [67]. It is important to conduct surveillance regularly in order to reduce the risk of the spread of pathogens [67]. Disease surveillance should be an integral and key part of all government aquatic animal health services [68].

#### 6.3.1. Passive Surveillance

Data collected for other propose can be utilized to know aquatic animal health status and to plan appropriate measures to reduce the incidence of disease. Data can be obtained from laboratories, field visits, research projects, from farmers, and aquaculturists. Passive surveillance is useful for early detection of emerging diseases. Its limitation is that it does not allow estimation of disease incidence and prevalence and it cannot be used to demonstrate freedom from disease [67].

#### 6.3.2. Active Surveillance

Active surveillance involves surveys to know the status of a particular disease in question. Evidence of disease in a specified population, and, in some instances, provides the data to prove that the specified population is free of a specific disease. Results of active surveillance may be biased unless properly designed and analyzed. Appropriate analyses can provide valid measures of incidence and prevalence of disease in particular area [73]. Its advantages include information better in quality, it is faster and cheaper to collect information than passive surveillance [74].

## 7. Importance Diagnostic Tests in Prevention and Control of Infectious Disease in Aquaculture

Diagnosing aquatic animals by the clinical sign is almost impossible because fishes live in water and move fast that make them impossible to visualize closely and inspect them for any clinical deviations. This makes rapid and accurate diagnostic methods to be important for the prevention and control of infectious disease. Diagnostic tests for identification of fish disease include conventional microbiological, immunoserological, and molecular methods. Rapid and accurate molecular-based methods have become important diagnostic tools. Lateral flow immunoassays, DNA microarray, proteins, or glycans can also be immobilized on a solid surface of the microarray to probe different target molecules labeled with fluorescence [75]. In diagnosing disease of fish, the detection of the pathogen in a tissue sample is conducted by lethal sampling rather than detection of antibodies that are an indicator of a particular disease, but in case of high valued fishes like ornamental fish, nonlethal sampling is recommended [8].

Diagnostic tests are not expected to be 100 percent sensitive and specific. To avoid misclassification, diagnostic test protocols should be selected and interpreted based on their performance under the conditions of use. In the context of biosecurity programs, diagnostic tests are used to detect the emergence and follow the progression of infectious agents in fish stocks. There are four main biosecurity-related objectives for which diagnostic tests are commonly used: to demonstrate freedom from infection in an aquaculture for obtaining or maintaining infection-free certification, to screen fish before introduction to the receiving facility, to detect infected fish as early as possible during a quarantine period, and to confirm suspicious or clinical case [76].

## 8. Challenges in Prevention and Control of Fish Disease

In relative terms, it is fair to say that infectious disease control in aquaculture is more complicated than terrestrial animal disease control due to environment where fish lives, and the nature of the fish themselves because fish cannot be observed close enough like we do in terrestrial animal, the environment can facilitate disease transmission quickly, fish are not cached easily without stress, they often gather in groups, and disease is often difficult to detect and characterize [5]. The other important challenge is in diagnosing disease of fish; in terrestrial animal disease diagnosis, the individual animal is the unit of interest. The scenario is not the same in aquaculture disease investigation because of the nature of the water where fish lives. A disease can transmit quickly and the whole tank may be the source of disease to healthy stock. In this case, the unit of interest is not a single fish rather the whole tank need to be investigated and diagnosed. Samples should be collected not only from fishes but also from water to measure important characteristics like pH, soil bottom conditions, and turbidity which makes aquatic animal diagnosis complicated and challenging [76].

## 9. Conclusion

It is clear that aquaculture is a huge industry operating worldwide and growing rapidly. The sector has been facing many constraints and challenges which are sophisticated and multifaceted. Among these challenges, infectious diseases take the lion share causing billion-dollar loss annually. Therefore, problem planning prevention and control strategy based on globally accepted principles and locally applicable strategies are recommended. These strategies should focus on preventing the development of infection rather than treating diseased stocks. Generally, the use of a combination of immunoprophylaxis, biosecurity measures, and use of only legally approved antibiotics can result in ultimate health protection of fish in aquaculture.

## Figures and Tables

**Table 1:** 

Sn.	Vaccine name	Aquatic species vaccinated	Diseases prevented	References
(1)	Aeromonas salmonicida Bacterin	Salmonids	Furunculosis	[33]
(2)	Arthrobacter Vaccine	Salmonids	Columnaris disease	[34]
(3)	Vibrio Anguillarum-Ordalii	Salmonids	Vibriosis	[35]
(4)	Infectious Salmon Anemia Vaccine	Salmonids	Infectious Salmon Anemia	[36]
(5)	Yersinia Ruckeri Bacterin	Salmonids	Yersiniosis	[37]
(6)	Infectious Hematopoietic Necrosis Virus Vaccine	Salmonids	infectious hematopoietic necrosis disease	[38]
(7)	Edwardsiella Ictalurii Vaccine	Catfish	Edwardsiellosis	[39]
(8)	Flavobacterium Columnare Vaccine	Catfish	Columnaris disease	[40]
(9)	*Vibrio anguillarum*-Ordalii	Rainbow trout	Vibriosis	[35]
(10)	*Vibrio salmonicida *Bacterin	Salmonids	Vibriosis	[41]
(11)	*Vibrio anguillarum-salmonicida *Bacterin	Salmonids	Vibriosis	[41]
(12)	*Edwardsiella ictaluri *Bacterin	Catfish	Enteric septicemia	[42]
(13)	Spring viremia of carp virus	Common carp	Spring viremia of carp	[43]
(14)	Koi herpes virus (KHV)	Koi carp	Koi herpes virus disease	[44]
(15)	Biofilm and free-cell vaccines of *Aeromonas hydrophila*	Indian major carps	Dropsy	[45]
(16)	*Streptococcus agalactiae *(group B) vaccine	Tilapia	Streptococcosis	[46]
(17)	Betanodavirus	Grouper	Betanoda virus disease	[47]
(18)	Enteric Redmouth (ERM) vaccine	Salmonid	Enteric red mouth disease	[48]
(19)	Pasteurella vaccine	Salmonid	Pasteurellosis	[49]
(20)	Aeromonas hydrophila vaccine	Salmonid	Motile Aeromonas Septicemia	[50]
(21)	Carp Erythrodermatitis	Carp	Erythrodermatitis	[51]
(22)	Piscirickettsia salmonis vaccine	Salmonid	piscirickettsiosis.	[52]
(23)	Gaffkaemia vaccine	Lobsters	Gaffkaemia	[53]
(24)	Nodavirus vaccine	Seabass	Viral Nervous Necrosis	[54]
(25)	Pancreas disease virus vaccine	Salmonid	Pancreas Disease	[55]

## References

[1] FAO (2016). *The State of World Fisheries and Aquaculture, Contributing to food security and nutrition for all*.

[2] Kubečka J., Boukal D. S., Čech M. (2016). Ecology and ecological quality of fish in lakes and reservoirs. *Fisheries Research*.

[3] Rico A., Satapornvanit K., Haque M. M. (2012). Use of chemicals and biological products in Asian aquaculture and their potential environmental risks: A critical review. *Reviews in Aquaculture*.

[4] Leung T. L. F., Bates A. E. (2013). More rapid and severe disease outbreaks for aquaculture at the tropics: Implications for food security. *Journal of Applied Ecology*.

[5] Peeler E. J., Taylor N. G. (2011). The application of epidemiology in aquatic animal health -opportunities and challenges. *Veterinary Research*.

[6] Romero J., Feijoó C. G., Navarrete P. (2012). Antibiotics in aquaculture Use, abuse and alternatives. *Health and Environment in Aquaculture*.

[7] Kumar V., Roy S., Meena D. K., Sarkar U. K. (2016). Application of probiotics in shrimp aquaculture: importance, mechanisms of action, and methods of administration. *Reviews in Fisheries Science and Aquaculture*.

[8] Harikrishnan R., Balasundaram C., Heo M.-S. (2011). Fish health aspects in grouper aquaculture. *Aquaculture*.

[9] Gudding R., Gudding R., Lillehaug A., Evensen O. (2014). Vaccination as a Preventive Measure. *Fish Vaccination*.

[10] Dadar M., Dhama K., Vakharia V. N. (2016). Advances in Aquaculture Vaccines Against Fish Pathogens: Global Status and Current Trends. *Reviews in Fisheries Science & Aquaculture*.

[11] Plant K. P., LaPatra S. E. (2011). Advances in fish vaccine delivery. *Developmental & Comparative Immunology*.

[12] Adams A. (2016). *Fish Vaccines*.

[13] Gudding R., Van Muiswinkel W. B. (2013). A history of fish vaccination: Science-based disease prevention in aquaculture.. *Fish and Shellfish Immunology*.

[14] Lien S., Koop B. F., Sandve S. R. (2016). The Atlantic salmon genome provides insights into rediploidization. *Nature*.

[15] Earle G., Hintz W. (2014). New approaches for controlling saprolegnia parasitica, the causal agent of a devastating fish disease. *Tropical Life Sciences Research*.

[16] Gudding R. (2014). Vaccination as a Preventive Measure. *Fish Vaccination*.

[17] Pridgeon J. W., Klesius P. H. (2012). Major bacterial diseases in aquaculture and their vaccine development. *CAB Reviews: Perspectives in Agriculture, Veterinary Science, Nutrition and Natural Resources*.

[18] Craig S., Klesius P., Gudding R., Lillehaug A., Evensen Ø. (2014). Replicating vaccines. *in Fish Vaccination*.

[19] Shoemaker C. A., Klesius P. H., Evans J. J., Arias C. R. (2009). Use of modified live vaccines in aquaculture. *Journal of the World Aquaculture Society*.

[20] Lillehaug A., Gudding R., Lillehaug A., Evensen Ø. (2014). Vaccination Strategies and Procedures. *Fish Vaccination*.

[21] Lorenzen N., Lapatra S. (2005). Vacunas de ADN para peces de vivero. *Revue Scientifique et Technique de l'OIE*.

[22] Ballesteros N. A., Alonso M., Saint-Jean S. R., Perez-Prieto S. I. (2015). An oral DNA vaccine against infectious haematopoietic necrosis virus (IHNV) encapsulated in alginate microspheres induces dose-dependent immune responses and significant protection in rainbow trout (Oncorrhynchus mykiss). *Fish and Shellfish Immunology*.

[23] Cho S. Y., Kim H. J., Lan N. T. (2017). Oral vaccination through voluntary consumption of the convict grouper Epinephelus septemfasciatus with yeast producing the capsid protein of red-spotted grouper nervous necrosis virus. *Veterinary Microbiology*.

[24] Dhar A. K., Manna S. K., Allnutt F. C. T. (2014). Viral vaccines for farmed finfish. *VirusDisease*.

[25] Mweemba H., Mutoloki S., Øystein E., Gudding R., Lillehaug A., Evensen Ø. (2014). Non Replicating vaccines. *Fish Vaccination*.

[26] Vallejos-Vidal E., Reyes-Lopez F., MacKenzie S., Austin B., Newaj-Fyzul A. (2014). Immunostimulant Diets and Oral Vaccination In Fish. *Diagnosis and Control of Diseases of Fish and Shellfish*.

[27] Aly S. M., Albutti A. (2014). Antimicrobials Use in Aquaculture and their Public Health Impact. *Journal of Aquaculture Research & Development*.

[28] Austin B. (2017). Antibiotics and Disinfectants. *in Diagnosis and Control of Diseases of Fish and Shellfish*.

[29] Plumb J. A., Hanson L. A. (2011). *Health Maintenance and Principal Microbial Diseases of Cultured Fishes*.

[30] Rogers C., Basurco B. (2009). Antimicrobial agents in aquaculture: Practice, needs and issues. *CIHEAM*.

[31] Bentzon-Tilia M., Sonnenschein E. C., Gram L. (2016). Monitoring and managing microbes in aquaculture – Towards a sustainable industry. *Microbial Biotechnology*.

[32] Korostynska O., Mason A., Nakouti I., Jansomboon W., Al-Shammaa A. Monitoring use of antibiotics in aquaculture.

[33] Antipa R., Amend D. F. (1977). Immunization of Pacific Salmon: Comparison of Intraperitoneal Injection and Hyperosmotic Infiltration of. *Journal of the Fisheries Research Board of Canada*.

[34] Salonius K., Siderakis C., MacKinnon A. M., Griffiths S. G. (2005). Use of Arthrobacter davidanieli as a live vaccine against Renibacterium salmoninarum and Piscirickettsia salmonis in salmonids. *Developments in Biologicals*.

[35] Boesen H. T., Pedersen K., Larsen J. L., Koch C., Ellis A. E. (1999). Vibrio anguillarum resistance to rainbow trout (Oncorhynchus mykiss) serum: Role of O-antigen structure of lipopolysaccharide. *Infection and Immunity*.

[36] Caruffo M., Maturana C., Kambalapally S., Larenas J., Tobar J. A. (2016). Protective oral vaccination against infectious salmon anaemia virus in Salmo salar. *Fish and Shellfish Immunology*.

[37] Tatner M. F., Horne M. T. (1985). The effects of vaccine dilution, length of immersion time, and booster vaccinations on the protection levels induced by direct immersion vaccination of brown trout, Salmo trutta, with Yersinia ruckeri (ERM) vaccine. *Aquaculture*.

[38] Anderson E., Clouthier S., Shewmaker W., Weighall A., LaPatra S. (2008). Inactivated infectious haematopoietic necrosis virus (IHNV) vaccines. *Journal of Fish Diseases*.

[39] Wise D. J., Greenway T. E., Byars T. S., Griffin M. J., Khoo L. H. (2015). Oral vaccination of channel catfish against enteric septicemia of catfish using a live attenuated Edwardsiella ictaluri isolate. *Journal of Aquatic Animal Health*.

[40] Shoemaker C. A., Klesius P. H., Drennan J. D., Evans J. J. (2011). Efficacy of a modified live Flavobacterium columnare vaccine in fish. *Fish and Shellfish Immunology*.

[41] Hoff K. A. (1989). Survival of *Vibrio anguillarum* and *Vibrio salmonicida* at different salinities. *Applied and Environmental Microbiology*.

[42] Plumb J. A., Vinitnantharat S., Paterson W. D. (1994). Optimum concentration of edwardsiella ictaluri vaccine in feed for oral vaccination of channel catfish. *Journal of Aquatic Animal Health*.

[43] Emmenegger E. J., Kurath G. (2008). DNA vaccine protects ornamental koi (*Cyprinus carpio koi*) against North American spring viremia of carp virus. *Vaccine*.

[44] Dishon A., Ashoulin O., Weber E. S., Kotler M. (2014). Vaccination against Koi Herpesvirus Disease. *Fish Vaccination*.

[45] Azad I. S., Shankar K. M., Mohan C. V., Kalita B. (2000). Uptake and processing of biofilm and free-cell vaccines of Aeromonas hydrophila in Indian major carps and common carp following oral vaccination - Antigen localization by a monoclonal antibody. *Diseases of Aquatic Organisms*.

[46] Evans J. J., Klesius P. H., Shoemaker C. A. (2004). Efficacy of Streptococcus agalactiae (group B) vaccine in tilapia (Oreochromis niloticus) by intraperitoneal and bath immersion administration. *Vaccine*.

[47] Patel S., Nerland A. H. (2014). Vaccination against Diseases Caused by Betanodavirus. *Fish Vaccination*.

[48] Villumsen K. R., Neumann L., Ohtani M., Strøm H. K., Raida M. K. (2014). Oral and anal vaccination confers full protection against Enteric Redmouth Disease (ERM) in rainbow trout. *PLoS ONE*.

[49] Barnes A. C., Dos Santos N. M. S., Ellis A. E. (2005). Update on bacterial vaccines: Photobacterium damselae subsp. piscicida. *Developments in Biologicals*.

[50] Poobalane S., Thompson K. D., Ardó L. (2010). Production and efficacy of an Aeromonas hydrophila recombinant S-layer protein vaccine for fish. *Vaccine*.

[51] Evenberg D., de Graaff P., Lugtenberg B., Van Muiswinkel W. B. (1988). Vaccine‐induced protective immunity against Aeromonas salmonicida tested in experimental carp erythrodermatitis. *Journal of Fish Diseases*.

[52] Evensen Ø. (2016). Immunization strategies against Piscirickettsia salmonis infections: Review of vaccination approaches and modalities and their associated immune response profiles. *Frontiers in Immunology*.

[53] Stewart J. E., Zwicker B. M. (1974). Comparison of Various Vaccines for Inducing Resistance in the Lobster. *Journal of the Fisheries Research Board of Canada*.

[54] Vimal S., Farook M. A., Madan N. (2016). Development, distribution and expression of a DNA vaccine against nodavirus in Asian Seabass, Lates calcarifier (Bloch, 1790). *Aquaculture Research*.

[55] Skjold P., Sommerset I., Frost P., Villoing S. (2016). Vaccination against pancreas disease in Atlantic salmon, Salmo salar L., reduces shedding of salmonid alphavirus. *Veterinary Research*.

[56] Defoirdt T., Sorgeloos P., Bossier P. (2011). Alternatives to antibiotics for the control of bacterial disease in aquaculture. *Current Opinion in Microbiology*.

[57] Maqsood S., Singh P., Samoon M. H., Munir K. (2011). International Aquatic Research Emerging role of immunostimulants in combating the disease outbreak in aquaculture. *International Aquatic Research*.

[58] Sharifuzzaman S. M., Austin B., edition., Austin B., Newaj-Fyzul A. (2017). Probiotics for Disease Control in AquacultureShellfish, 1st. *Diagnosis and Control of Diseases of Fish and Shellfish*.

[59] Mastan S. A. (2015). Use of immunostimulants in aquaculture disease management. *Int. J. Fish. Aquat. Stud*.

[60] De B. C., Meena D. K., Behera B. K., Das P., Das Mohapatra P. K., Sharma A. P. (2014). Probiotics in fish and shellfish culture: Immunomodulatory and ecophysiological responses. *Fish Physiology and Biochemistry*.

[61] Hermosillo O. A. M., Mart P., Ib A. L., Ram H. C. (2012). Use of Probiotics in Aquaculture. *International Scholarly Research Notices*.

[62] Carbone D., Faggio C. (2016). Importance of prebiotics in aquaculture as immunostimulants. Effects on immune system of Sparus aurata and Dicentrarchus labrax. *Fish and Shellfish Immunology*.

[63] Reverter M., Bontemps N., Lecchini D., Banaigs B., Sasal P. (2014). Use of plant extracts in fish aquaculture as an alternative to chemotherapy: current status and future perspectives. *Aquaculture*.

[64] Miriam R., Tapissier N., Pierre S., Saulnier D., Austin B., Newaj-Fyzul A. (2017). Use of Medicinal Plants in Aquaculture. *Diagnosis and Control of Diseases of Fish and Shellfish*.

[65] Awad E., Awaad A. (2017). Shell fish Immunology Role of medicinal plants on growth performance and immune status in fish. *Fish and Shellfish Immunology*.

[66] Phu T. M., Phuong N. T., Dung T. T. (2016). An evaluation of fish health-management practices and occupational health hazards associated with Pangasius catfish (Pangasianodon hypophthalmus) aquaculture in the Mekong Delta, Vietnam. *Aquaculture Research*.

[67] Oidtmann B. C., Thrush M. A., Denham K. L., Peeler E. J. (2011). International and national biosecurity strategies in aquatic animal health. *Aquaculture*.

[68] Hadfield C. A., Clayton L. A. (2011). Fish quarantine: Current practices in public zoos and aquaria. *Journal of Zoo and Wildlife Medicine*.

[69] Jia B., St-Hilaire S., Singh K., Gardner I. A. (2017). Biosecurity knowledge, attitudes and practices of farmers culturing yellow catfish (Pelteobagrus fulvidraco) in Guangdong and Zhejiang provinces, China. *Aquaculture*.

[70] Adams A., Thompson K. D. (2006). Biotechnology offers revolution to fish health management. *Trends in Biotechnology*.

[71] Taw N. (2017). Biosecurity in Aquaculture Systems with Special Emphasis on Shrimp Farming. *Journal of Fisheries & Livestock Production*.

[72] Dvorak G. (2009). Biosecurity for Aquaculture Facilities. *North Central Regional Aqua culture Center*.

[73] Scarfe A. D. (2006). *Aquaculture Biosecurity Prevention, Control, and Eradication of Aquatic Animal Disease*.

[74] Landman M. J., Ling N. (2011). Fish health changes in Lake Okaro, New Zealand: Effects of nutrient remediation, season or eutrophication?. *Hydrobiologia*.

[75] Kim A., Nguyen T. L., Kim D.-H. (2017). Modern Methods of Diagnosis. *Diagnosis and Control of Diseases of Fish and Shellfish*.

[76] Ninawe A. S., Hameed A. S. S., Selvin J. (2017). Advancements in diagnosis and control measures of viral pathogens in aquaculture: an Indian perspective. *Aquaculture International*.

